# Stories told by plants on graveyards in Northern Angola

**DOI:** 10.1371/journal.pone.0236941

**Published:** 2020-08-17

**Authors:** Thea Lautenschläger, José Lau Mandombe, Monizi Mawunu, Christoph Neinhuis

**Affiliations:** 1 Institute of Botany, Department of Biology, Faculty of Science, Technische Universität Dresden, Dresden, Germany; 2 University of Kimpa Vita, Province of Uíge, Uíge City, Angola; Ilia State University, GEORGIA

## Abstract

**Background:**

Worldwide, different traditions of symbolic statements in graveyards can be found. However, studies on sub-Saharan Africa are rare. For BaKongo cemeteries, it is only known that they traditionally do not exhibit plants for decoration purposes. Our study wanted to inspect the influence of Portuguese culture due to the long shared colonial past.

**Methods:**

During 2015 and 2019, plant use in 87 graveyards in 13 municipalities of the province Uíge was documented. Five expert interviews with the village eldest in five municipalities completed the data collection.

**Results:**

While 24% of the graveyards didn´t have any planting, 27 plant species were found in the remaining ones, including a high percentage of alien species (59%), mainly from the Americas. The most abundant plant species are *Euphorbia tirucalli* (23%) and *Agave sisalana* (22%). With increasing distance from the city Uíge (especially towards the Democratic Republic of the Congo), the utilization of living plants in cemeteries is decreasing except along the road. In most of the cases, just one plant species per gravesite was found.

**Conclusions:**

This unexpected high number of plants might be interpreted as a strong evidence of outside influence. Cultural symbols of the BaKongo cosmology and Christianism appear to coexist or coalesce. Furthermore, plants are used as a marker for graveyards. Modern influences like the use of concrete in proximity to urban areas indicate a certain wealth.

## Background

**Tufwanga mu soba–Dying is not the end**. **(KiKongo proverb)**

Virtually every culture worldwide reserves a specific space for their deceased. These graveyards and associated symbolic statements differ with respect to cultural characteristics and the traditions resulting thereof. Concurrently, cemeteries reflect changes in society introducing new types of burials such as collective graves for AIDS victims or soccer fans, reflecting ideological developments as well [1]. But also regional vegetation units as well as the symbolism attributed to selected plants played a major role in graveyard arrangement and decoration [2].

The magic of a plant may originate from a remarkable growth form, shape, scent or color, the function in its habitat, relation with animals, an associated medicinal use, or in its connection to ancestors [3,4]. For England, Drury (1994) [5] describes a wide spectrum of traditions and customs which prevailed from early times such as the use of garlands as well as the strewing of flowers, herbs and evergreen on graves. The long lasting green of species like *Buxus sempervirens*, *Rosmarinus officinalis*, *Taxus baccata* or *Laurus nobilis* symbolizes immortality, especially those exhibiting extreme longevity. Aromatic herbs with a high content of aromatic volatiles became widely used to hide the smell of the dead before burial. The 19^th^ century introduced the fashion of decorating graves with a variety of flowers the colors of which allegorize different meanings. Hohla (2003) [6] added *Hedera helix* as one of the most widely used plants on German cemeteries, symbolizing everlasting fidelity and thirst for eternity that may have originated from its leaning and entwining growth, keeping faith with the entwined object even after its death [7]. *Calendula officinalis* was called „the plant of the dead”and therefore planted on graves. By contrast, *Thuja* and *Cupressus* were regarded as “trees of life” and thus served as bridge into the next world [6].

Traditions of graveyard and cemetery decoration in Mexico are described by Castro (2001) [8]. Fresh or plastic flowers are placed on the grave, in combination with crosses made of wood or molded concrete. Fences of different material enclose the gravesites. Grave markers are often painted colorfully, sometimes including religious motives.

The therapeutic potential has been proven for several sacred plants [3,9–11]. An interesting study from Pakistan detected graveyards as spots of increased biodiversity and an essential source for medicinal plants as they are the least disturbed areas due to the religious beliefs of the local people. As a consequence, graveyards should be conserved to protect natural resources for the benefit of humankind [12].

The review of Dafni et al. [13] about the ritual plants of Muslim graveyards in northern Israel illuminates Israel’s unique geographical position and called it a “crossroad in the cultural arena between Asia and Europe” due to the mixture of plants used and their original symbolism. The authors detected three groups of plants: aromatic herbs (especially *Salvia fruticosa* and *Rosmarinus officinalis*), white flowered plants (mainly *Narcissus tazetta*, *Urginea maritima*, *Iris* spp. and *Pancratium* spp.) and *Cupressus sempervirens* as the most abundant graveyard tree and assumed that the use of white flowers as cemetery plants reflects an old European influence. A very similar pattern also arose from the planting of trees and shrubs in Muslim cemeteries in Israel as they are similarly used in ancient as well as in modern European cultures [13].

Although the Association of Gravestone Studies [14] provides a large number of studies on gravestones and their (botanical) symbols worldwide, studies on sub-Saharan African cemeteries are rare, especially those concerning the use of plants. However, several ethnological studies list the scientific names of the mentioned plants [15]. The increasing number of historical and present studies on Afro-American cultures provide an important link because the slave trade, especially originating in West Africa, transferred not only workers but also their traditional knowledge of plants as well as their cultural background and customs to the New World. These elements became part of ceremonies in newly-evolving religions like Candomblé in Brasil or Santería in Cuba [16,17]. In the book “African Ethnobotany in the Americas”, composed of 14 articles [18], the authors explain multiple levels of relationship between plants and people. Inter alia, ritual plants of the Surinamese Winti culture are documented [3]. Within the 411 recorded plant species, statistically significant plant features for the spirit type “deceased” are spines and itching hairs, blue color and a bad smell, used mainly to calm down the angry ghost of the deceased, while for the spirit type “ancestors” statistical evidence for cultivated food plants were found [3].

Although North America, received only half a million Africans during the entire slave trade [19], influences of traditional plant use on the early slave cemeteries in southern plantations of the United States were observed [20]. Besides the small markers made from stone or wood, graves often were decorated with personal objects such as cutlery, mirrors or bottles to accompany the decedents on their journey into the afterlife as well as to keep away evil spirits. Vlach (1990) [21] observed these decorations as well and argued that graveyard decoration represents a spiritual continuity between Africa and its people in the diaspora. Bolton (1891) [22] already had made similar observations on graves in South Carolina, lacking the understanding of the cultural background, until he read the travelogue of Glave (1892) documenting his voyage to the Congo. Already in 1892 the English adventurer Edward James Glave described the decoration of the graveyard of a Congo chieftain in his book *In savage Africa* [23] leading to the question how the Kongo cultural heritage was transferred to the New World.

There are just a few descriptions of cemeteries or burial practices in Angola. Lopes Cardoso (1962) [24] describes the comparatively young *mbali* funeral art from the south of Angola. The heterogeneous origin of the *mbali* people and the European influence is reflected in the carved crucifixes made of wood, stone or even concrete and the illustration of the deceased. Martínez-Ruíz (2013) [25] depicts an imbondeiro tree (*Adansonia digitata*) in a BaKongo cemetery of elders thirty-five kilometers north of Mbanza Kongo that’s shows several engravings like triangles and lines. Mbanza Kongo was the capital of the former Kongo Kingdom. The Kongo Kingdom, a Bantu empire that existed between the 14^th^ and 18^th^ century, was located in an area that is spread over four countries today–from Gabon in the north to the Republic of the Congo, the western portion of the Democratic Republic of the Congo (DRC) and northern Angola in the south. It had a centralized government, a national currency, and well-developed markets and trading networks, which was favorable for the trade with Europeans [26]. Nevertheless, it was far from being homogeneous because several adjacent peoples were assimilated, different dialects were spoken [27]. In the area of present-day Angola’s´ Northern Province Uíge alone, eleven ethnic subgroups of BaKongo are to be found [28].

In several letters written by King Afonso I of the Kongo Kingdom (1509–1543) to the Portuguese kings he distinguished between so called “nossos filhos, parentes e naturae” (our children, relatives and natives [citizens]) and “espriuos/espravos”(slaves), who at the beginning of the trade mostly were prisoners of war [29]. As the latter had monetary value in Portugal he was using the slaves as commodity exports. Slave trade markets were supported and therefore until the eighteenth century, slaves covered international financial and even diplomatic obligations.

A comprehensive work on the BaKongo north of the Congo River at the turn of the 19^th^ century was published by the Swedish missionary Karl Laman, although the accuracy is debatable due to an confusing editorial policy and the existence of several translations [30]. Nevertheless, the chapter “Death” in his four-volume ethnography *The Kongo* describes plenty of rituals and restrictions accompanying burials. The decoration of the graves is limited to personal things like umbrellas or porcelain figures and household utensils like plates with holes knocked in the bottom to prevent them from being stolen. Plants for grave decorations are not mentioned but several rituals are described, where plants play an essential role. Laman (1957) [31] describes that before burials hair, nail-parings and the dirt from the body of the deceased were put into a palm-kernel in which a hole has been gnawed by the ngoni-rat and placed under the nsambvi-tree. While the bodies of rich people are wrapped with clothes poor people are shrouded in a papyrus mat (*Cyperus papyrus*) before lowering the body into the grave. Stalks of the matutu-grass are placed around the grave to protect the deceased from the bandoki (witches) as the stalks imitate guns. Several medicines are described to be used for the deceased: nkandikila, lungungu lwa nsamba, luyalu and luvemba, mainly with the purpose of shielding and providing support. Also, the time of mourning is sometimes determined by palms. Tall palms are cut down and used as firewood for the mourners. The period of weeping ends when the palm has been consumed.

A similar situation is reported by the German explorer Adolf Bastian in his book “Die deutsche Expedition an der Loango-Küste” (1874) [32]. Beside the earth graves with their typical cookware decoration and wooden constructions on the graveyards for burial ceremonies, he also mentioned a tree called *Sandä*, planted at the graves. This is probably *Ficus thonningii*, in Uíge called *n´sanda* and of high cultural importance in the area. Furthermore, he described a tree called kattu-sankondo that was used only for the right arrangements of the dead bodies of priest-kings [33].

Likewise, Güssfeldt et al. (1888) [34] and Pechuël-Loesche (1907) [35], traveling along the Loango coast from 1873 to 1876, mentioned corpses rolled up in typical papyrus mats and hanged in a horizontal position as well as perforated pots and other kitchen devices on the burial mounds. Not a word on the use of plants or flowers at the cemeteries. On the contrary, when the author once threw some flowers on the grave people took it away quickly: “„Das wäre nicht Brauch, bedeutete er mich, und könnte die Toten stören,”[35] [This is not tradition, he said, and could disturb the dead.”].

To understand Kongo people and their position within the cosmos, the dikenga dia Kongo (the Kongo cosmogram) is an important although very complex source of information [2,36,37]. This cosmogram is sometimes strongly reduced: two lines crossing form a simply form of the dikenga to give rise to a spiritual connection [38]. Interestingly the Catholic concepts like heaven, hell or the symbolism of water (BaKongo: spirit world; Christianism: baptism) were absorped by the BaKongo. In the mid seventeenth century, wooden crucifixes, also two lines crossing, became a common adored *nkisi*, an object of spiritual power. Pechuël-Loesche (1907) [35] described for the Loango-coast the use of a cross inside a circle as a symbol for forks, traditional places of jurisdiction.

Angola´s tragic recent history including 12 years of independence and later 28 years of civil war caused considerable refugee movements to the DRC. Therefore, traditional village structures were often destroyed, especially in the country’s north. Nevertheless, some customs are still continued such as the planting of a cutting of N´sanda (*Ficus thonningii*) on the occasion of the foundation of a new village [39,40], those roots are reaching all the houses and thus connecting all inhabitants (pers. comm.), or the welcome gift including the three components, cola nut (*Cola acuminata*), hot pepper (*Capsicum* sp.), and salt [41]. During ceremonies like funerals, palm wine, mostly made of *Raphia matombe*, is offered to the guests (Monizi et al., 2018). This study takes a closer look only at the funeral symbols, especially plants, recently documented in the different municipalities of Angola’s Northern Province Uíge to discuss the impact of Christianization on people’s funeral places.

## Materials and methods

### Study area and population

Field work was carried out in Angola´s northern Province of Uíge, bordering in the north and east on the Democratic Republic of the Congo, in the south on the provinces of Malanje, Cuanza Norte, and Bengo, and in the west on Zaire province. Uíge covers an area of about 60,000 km^2^ between 6 and 8 degrees latitude south and 14 and 17 degrees longitude. While most of the country is characterized by different savannah formations and even deserts, sometimes including gallery forests, the north of the country represents a mosaic of forest remnants and regularly burned savannahs exhibiting both tropical rainforest and savannah species comparable to the Bas-Congo region in the Democratic Republic of the Congo [42–44]. Nearly all habitats show severe anthropogenic disturbance, mainly due to logging, slash and burn agriculture as well as uncontrolled savannah fires [45].

The Province houses over 1.4 Million people (CENSO 2014) the majority belonging to the KiKongo speaking BaKongo. This Bantu ethnic group nowadays is distributed over the four countries Angola, Republic of the Congo, Democratic Republic of the Congo, as well as Gabon. During the time of the formerly known *Kongo dya Ntotila* (Kingdom of Kongo) whose capital M´banza Kongo was located in the in the territory of present-day Angola, a huge cultural diversity was and up till now is present including a wide range of traditions. Eleven ethnic BaKongo-subgroups are present in the province: Muzombo, Bacongo, Maiaca, Sosso, Muxicongo, Mahungo, Puna, N´gola, Ginga, Pombo, and Massuco [28].

### Data collection

Embedded in another ethnobotanical study [41], the sampling took place in 13 municipalities between July 2015 and November 2019. In total, 87 graveyards were visited, of which only a small proportion (17%) was situated on the main road. We did not create a species list of each gravesite, but for each cemetery. Furthermore, we conducted five interviews with the village eldest to obtain information about plant use and its symbolic meaning as well as about funeral practices. These interviews took place in five different municipalities (Damba, Kangola, Negage, Songo and Uíge). We used the topic list containing 20 questions (S4) as a guideline to implement semi-structured-interviews [46]. While in Damba, Negage and Uíge only one male interviewee was asked, in Kangola and Songo a group of three to five male interviewees were questioned, all aged 40+. The confirmation of the age of the informants was difficult due to missing or falsified identification documents (to prevent being drafted to army during the long military conflict). In preparation, the University Kimpa Vita formulated credentials to inform the mayors of the municipalities about the planned activities that were included in a large-scale ethnobotanical survey. To establish contact with potential informants, local authorities of the visited villages (called *soba* and *seculo*) were informed about the aims and methods of the study and asked to suggest persons that might be willing to participate (prior informed consent) in the study. The soba of each village was always part of the interviewed group. During a visit of the village cemetery, we documented vernacular plant names in KiKongo as well as information about growth form and vegetation units. During fieldwork, the languages Portuguese and KiKongo were used. The local authorities permitted and accompanied the whole survey process. We followed the code of ethics of the International Society of Ethnobiology. The study was carried out in compliance with the agreement of Access and Benefit Sharing. For identification, plants were photographed and plant voucher specimens were collected, dried and stored at the Herbarium Dresdense (DR), Technische Universität Dresden, Germany. In a Memorandum of Understanding, signed in 2014, the Instituto Nacional da Biodiversidade e Áreas de Conservação (INBAC), Angola and the Technische Universität Dresden, Germany agreed that duplicates will be returned to Angola as soon as appropriate conditions to store the herbarium vouchers are established. The Ministry of Environment Angola and the Provincial Government of Uíge issued the required collection and export permits. Plant species on the grave sites as well as in the closer area were documented and collected. Identification of these plant specimens and data analysis was completed in Dresden, Germany. For identification, the Flora of Angola, Flora Zambesiaca, and Flora of West Tropical Africa were used. Additional information was retrieved from Kew Herbarium Catalogue [47] and Naturalis Biodiversity Center [48]. The Herbaria in Lissabon (LISC) and Coimbra (COI) were visited in July 2016 and 2017 for comparing plant samples [49]. Kew´s Plantlist [50] was used as the basis for the applied nomenclature. Voucher specimen numbers of Herbarium Dresdense as well as photo voucher numbers are given in S1 Table. The checklist for Angola (54) helped to identify alien species, i.e. plant species that are not native to a certain geographical region, here to Africa. Later, we added the origin of these neophyte species. Furthermore, we listed and compared the medicinal uses according to Lautenschläger et al. (2018) [51], corresponding plant families, growth forms, number of cotyledons, as well as characteristic colours of the striking features or iterative growth patterns of the plant. The Chi-square test of independence was used to determine whether a significant relation between two variables exists.

## Results

### General aspects

**Fig 1:**
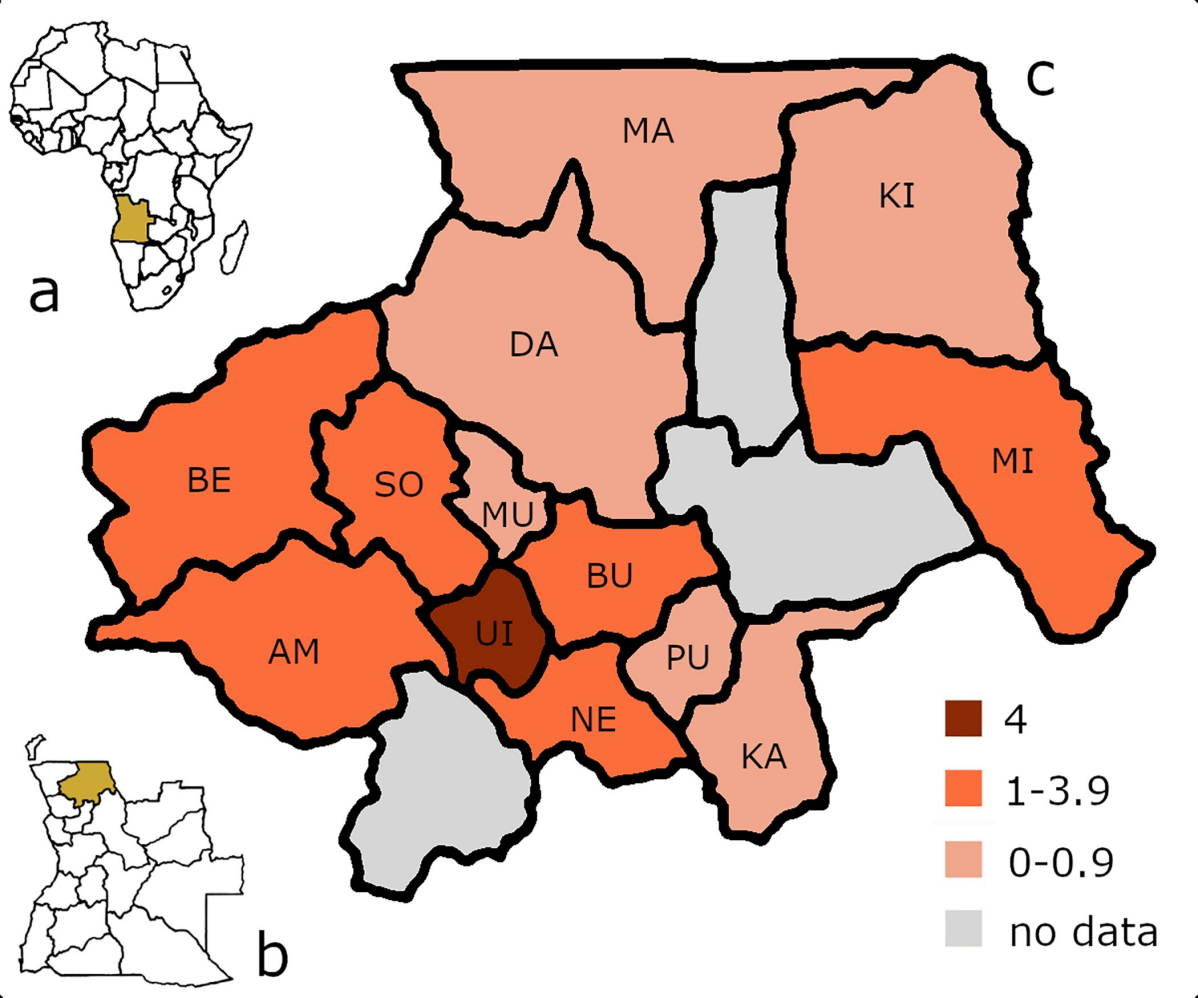
Average plant species number. (a) Location of Angola in Africa (map based on https://landlook.usgs.gov/landlook/viewer.html), (b) Province of Uíge in Angola (map based on https://commons.wikimedia.org/wiki/File:Covid19-angola.jpg), (c) Average number of plant species used in cemeteries in the different surveyed municipalities (map based on https://commons.wikimedia.org/wiki/File:Angola_municipalities.png).

**Fig 2:**
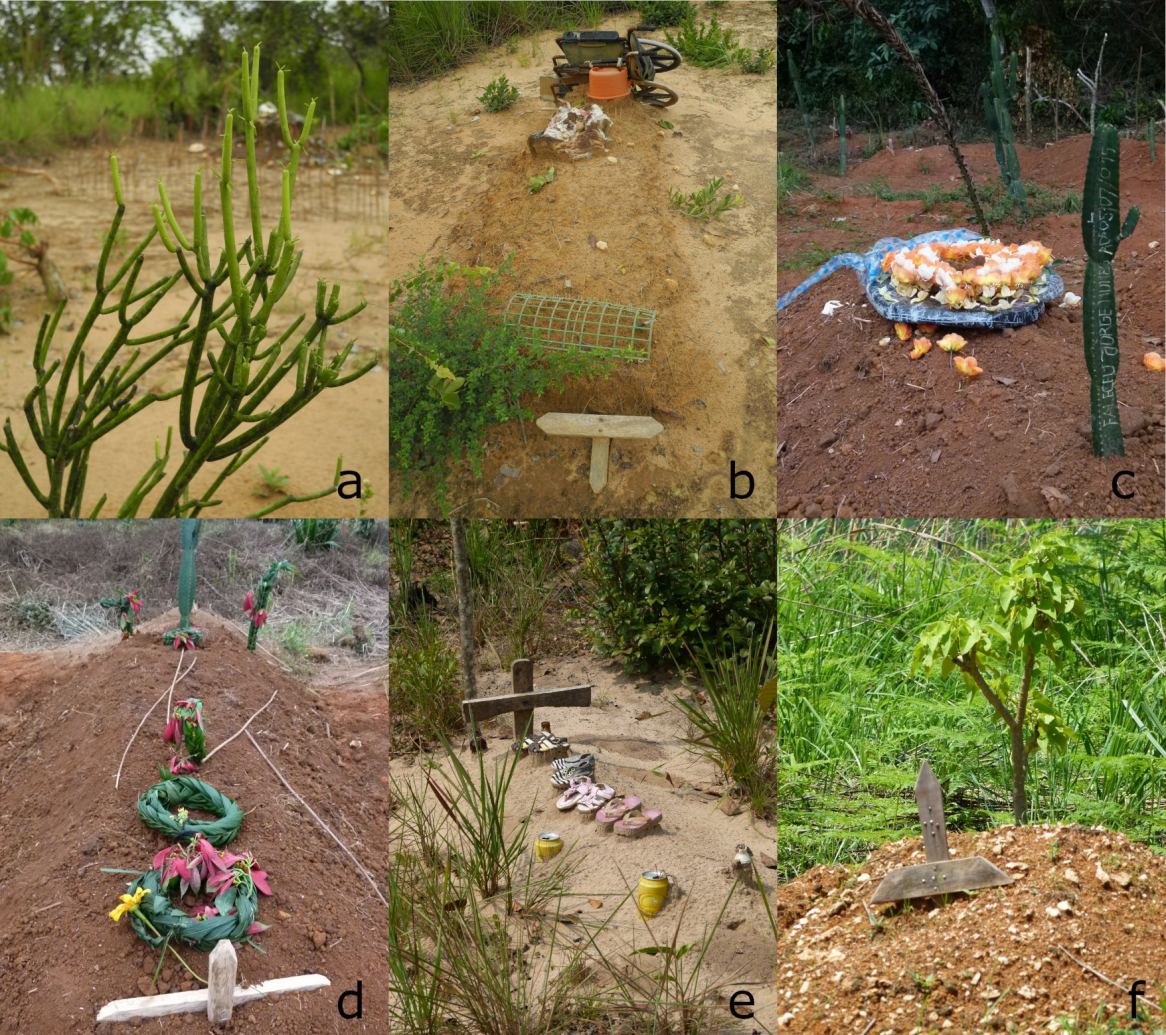
Plants on graveyards in Uíge province. (a) *Euphorbia tirucalli*, the succulent stems branch in regular intervals, in the background another BaKongo graveyard, (b) Graveyard of a handicapped man, decorated with his wheelchair, (c) *E*. cf. *ingens* and artificial flowers, (d) garland made of palm leaves and flowers of *Euphorbia pulcherrima* and *Tithonia diversifolia*, (e) Children’s shoes, (f) *Jatropha curcas* and a wooden cross with nails probably showing elements of the BaKongo cosmogram.

**Fig 3:**
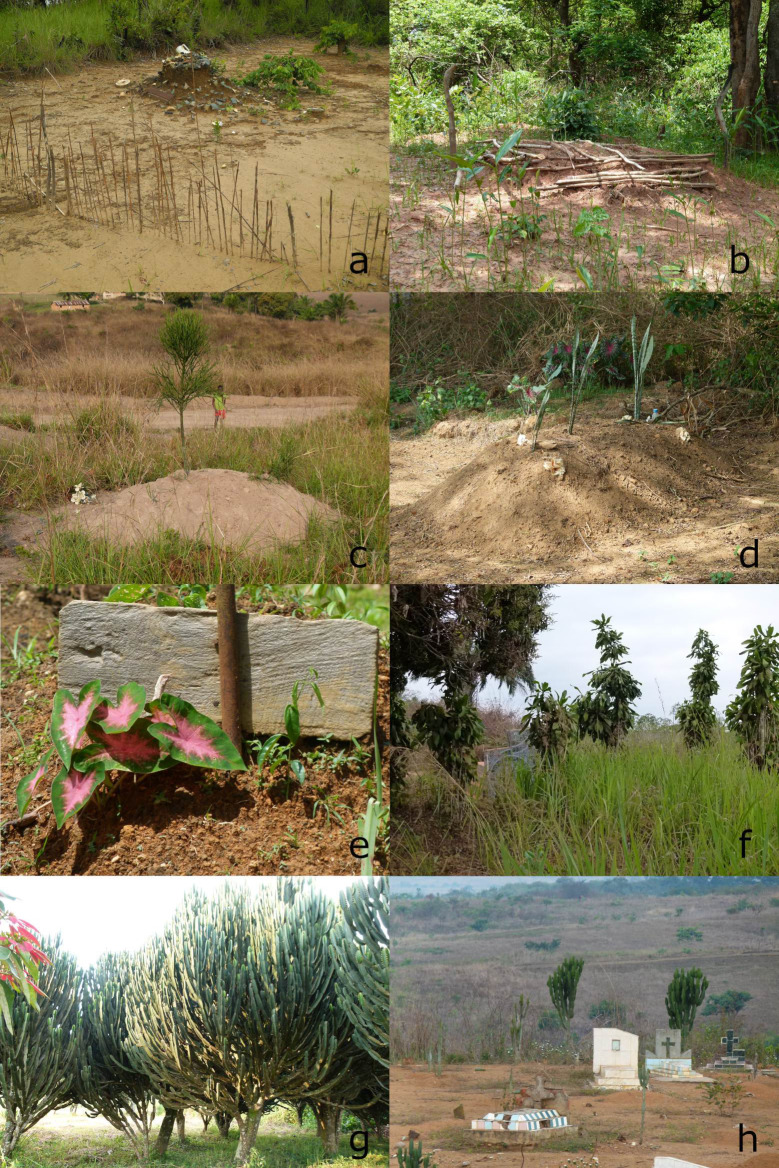
Plants on graveyards in Uíge province. (a) Traditional BaKongo graveyard with old ceramic, (b) *Erythrophleum africanum* used for mark graveyards in the municipality of Kangola, (c) *Euphorbia tirucalli*, (d) Sansevieria sp., (e) *Caladium bicolor*, (f) *Dracaena fragrans*, (g) *Euphorbia* cf. *ingens* trees, (h) hudge *Euphoria* trees at a cemetery in the municipality of Bembe.

In our study, 24% of the 87 graveyards visited didn´t have any plantings. With increasing distance from the city Uíge (especially towards the DRC) the utilization of living plants in cemeteries is decreasing except along the road. Fig 1 shows the average number of plant species used in the different surveyed municipalities. In the municipality Uíge with the capital Uíge, in average four species per graveyard are used. In the surrounding municipalities, in average 1 to 3.5 plants were found, while in the remote municipalities, less than one species is planted. In Milunga, we only visited one cemetery. Therefore, the comparatively high number (2) might not be meaningful. In most cemeteries, one species is the dominant one. The most abundant plant species in cemeteries are *Euphorbia tirucalli* L. (23%) and *Agave sisalana* Perrine (22%). While *Agave sisalana* is spread all over the province, *Euphorbia tirucalli* has its main occurrence in graveyards in the municipalities Damba and Maquela do Zombo in the north of Uíge city towards DRC (Fig 2A). In the areas of Uíge and Negage *Dracaena fragrans* (L.) Ker Gawl. and *Euphorbia pulcherrima* Willd. ex Klotzsch are abundant. *Euphorbia* cf. *ingens* E.Mey. ex Boiss. and *Jatropha curcas* L. are common in cemeteries of the whole province. Besides the eye-catching green shrubs and trees, a selected number of rather small flowering plants were found such as *Catharanthus roseus* (L.) G.Don. Furthermore, the prominent inflorescences of agaves indicate the presence of graveyards from afar. Dry sticks of *Erythrophleum africanum* (Benth.) Harms are used in the savannah woodlands of the municipality of Kangola to mark the limits of graveyards (Fig 3B). In this municipality, living plants are very rare. Overall, the highest absolute diversity of plant species was found in Uíge (16 species), Negage and Songo (11 species each).

Moreover, other elements are used to decorate the gravesites, for adults as well as for children, especially on graveyards without any plantings. Burial gifts often have a personal connection to the deceased, such as shoes (Fig 2E) or a ritual value such as beer cans. If small babies died, their feeding bottles were placed on the graves. A school boy´s backpack and pencils adorned his grave. A handicapped person´s wheelchair decorates the site (Fig 2B). Artificial flowers of various colors and form are increasingly found as well but we never found shells on the graveyards (Fig 3C).

Two plant families seem to be most important: 25% of the species are Euphorbiaceae, 17% Asparagaceae. The five most abundant plants belong to these two families.

### Color and iterative growth patterns

Plants are used for decoration mainly due to their colorful flowers such as *Tithonia diversifolia* (Hemsl.) A.Gray, *Canna indica* L., *Amaryllis* and *Lantana camara* L. or colorful leaves such as *Caladium bicolor* (Aiton) Vent. (Fig 3E). Assessing the colors of the 27 plant species, some patterns became evident. 42% were just green, followed by the colors red (29%) and yellow (27%). White color was found in 5 species (19%) and blue just in one (4%). 50% of the color features were provided by leaves or stems (green), 38% by the flowers and in just of one species the red fruits were used for decoration (*Alchornea cordifolia* (Schumach. & Thonn.) Müll.Arg.). Most of the “green” species, representing six out of the seven most abundant species, show a peculiar iterative or spiral growth instead of color. Species with spiral growth forming a rosette are *Agave sisalana*, *Cordyline fruticosa* (L.) A.Chev., *Dracaena fragrans* and *Elaeis guineensis* Jacq [52]. Here, the leaves are clustered tightly together. Iterative growth patterns are characterized by self-similar branching of highly similar segments. For example, in the most abundant species, *Euphorbia tirucalli*, the succulent stems branch at regular intervals (Fig 2A).

### Alien plants

A high number of documented species (59%) is not native to mainland Africa. 14 of these 16 species originated from Central- or South-America; one species was introduced from Asia (*Cordyline fruticosa*) and one from Madagascar (*Catharanthus roseus*). No significant difference between growth form and origin could be detected (chi-square test, P > 0.05). Even if we list growth forms here (S1 Table), the data should be read with care without any overvaluation because in some cases, no clear classification is possible as several species can vary between tree or shrub form (for example *Alchornea cordifolia* or *Euphorbia ingens*). No significant differences between origin and plant family or origin and medicinal use was found (chi-square test, P > 0.05).

### Interviews

#### Plant use

Depending on the investigated municipality, different plants are used and therefore mentioned by the interviewees. The following declarations are based on these statements. Vernacular names are found in S1 Table. In any case, plants with colorful leaves or flowers were planted due to their beauty and therefore “spring from heart”, as people said. *Euphorbia* cf. *ingens* and *E*. *tirucalli* were mentioned as grave markers and as very resistant to wind and fire. *Dracaena fragrans* does not need much care as well and in former times was planted very often to show the limit of a certain area (Fig 3F), which also accounts for *Agave sisalana*, *Sansevieria* sp., *Newbouldia laevis* (P.Beauv.) Seem. ex Bureau and *Jatropha curcas*. According to the respondents, the two neophytes *Senna occidentalis* (L.) Link and *Canna indica* were not planted by purpose but appeared accidentally. Both were found twice supporting this statement. A spiritual or religious use was never mentioned nor that a plant would bring good fortune. Solely *Euphorbia* cf. *ingens* and *Cereus* sp. were denominated to serve a function as a lightning conductor as both were observed to be struck by lightning.

According to the statements of the respondents, the high social status of a person is indicated by having tiled gravestones without any plants while for decedents with a lower social status, plants are often used. *E*. cf. *ingens* stems being comparatively thick and having a persistent periderm are used as living gravestone by engraving the name of the decedent on them. Since wooden crosses do not last very long they are occasionally replaced by crosses made of molded concrete. Crosses often are located above the head of the buried person. The same applies for *Euphorbia* cf. *ingens* used as marker, and for *Newbouldia laevis* while other plants could also be planted in the middle or the opposite end of the grave. Most of the species are planted on the grave during the burial ceremony, rarely some days later. The planting itself did not involve a special ritual, song or prayer. Although most of the people are Catholic or belong to a Protestant Free Church, many gravesites do not show any cross or other symbol. The interviewed soba of Maquela do Zombo said that Christians are buried with a cross on their grave while the gravesites of non-Christians are decorated by a cutting of *Newbouldia laevis*. No differences are made between men and women nor are any different procedures carried out for minorities like twins, priests, sick persons or those that committed suicide. During one interview, it was mentioned that murderers are not allowed to have their gravesite in the limits of the graveyard.

Cemetery maintenance varies according to the organization structure within a village. While In some villages the area is cleaned regularly, this is done just once a year in others. Interestingly, the responsibility for planting and maintenance of the graves varied from “the family” to “everybody in the community if the family is not living in the village” to “nobody–it is just left to nature”. While some of the graveyards show a structured arrangement with an entrance made of stones or concrete, the majority do not. Cemeteries are always located outside or beside villages–meaning that the dead inhabit their own village adjacent to the living to rest in peace, but not that far away. Around the villages, the dominant habitats are anthropogenically influenced savannahs characterized by regular savannah fires that also penetrate the cemeteries. Therefore, *Euphorbia tirucalli* and *E*. *ingens*, resistant to frequent fires and wind, are preferentially used. Furthermore, most species can be easily propagated by cuttings so that existing plants serve as source for new plants.

The interviewees themselves believe that the ancestors reside in their own land near the graveyards. The oldest of the village are responsible for taking care of the ancestors–an important task as the ancestors’ power corresponds to those of living parents [53].

According to the answers received, plants like *Agave sisalana* or *Euphorbia* species may indicate the presence of a currently used or former cemetery. But, because the plant species are also used as living fences this is not necessarily the case. Asking people whether they avoid places with one or several of the mentioned plants the answers ranged from “no” to “it could be a cemetery and therefore better not to cross” and avoid the way. One person believed that it depends on the tribes–so for some it could symbolize a “bad atmosphere”. However, due to the refugee movements of people to the DRC and nowadays back to Angola during wartimes, traditions were mixed a lot, and hence, plant use too.

Urbanization and colonial influence introduced other patterns of placement of cemeteries. Under Portuguese influence, cemeteries were opened alongside churches. As an example, the main cemetery of the capital of the Province Uíge was established by the Portuguese during the colonial era and reopened in 2003 by the Minister of Youth and Sports. Due to the age of the cemetery some of the tree specimens are already old, especially individuals of *Pinus* and *Callitris*, the use and occurrence of which in Angola was already described by Gossweiler in 1950 [54]. The 3.3 hectares site is partly untended and contains graveyards with and without plants, depending on the family. Nearly all plants listed in S2 Table are to be found.

#### Rituals

Funeral rites include weaving of palm leaves as entrance portals to the cemetery, sometimes additionally decorated with flowers (*Tithonia diversifolia*) and fruits (*Alchornea cordifolia*), as we could see during our inspections. Furthermore, palm leaves are used to bind garlands or to decorate the graves. Palm fronds also serve as symbols on cars used for transportation of sick or dead people.

In Angola, at the occasion of the 2^nd^ of November, called *Day of Decedent* (Dia de Defunto, Dia dos Finados), people maintain cemeteries all together, by cutting the large savanna grass (locally called capim) or rebuilding the graveyards suffering from erosion. According to the interviewees, plants are helpful to locate the graveyards because the plants differ from the surrounding vegetation. This may be explained by the fact that 59% of the species are not native to mainland Africa. As people of the villages know about the plant use on the graves, they use them as a mark. Each family decides from themselves whether they use a plant marking and which one.

Furthermore, rituals are carried out involving the spreading of cola-nut (*Cola acuminata*) and palm wine (*Elaeis guineensis* or *Raphia* sp.) mixed with healing soil (that normally is used for medical treatments in terms of geophagy) over the cemetery. During his journey through the area south of the Congo River in January 1886, at that time called São Salvador, the German explorer Richard Büttner [55] attended such a cleaning of a burial ground. A hail of bullets initiated the day. Women like men and children cut the grass using machetes until the ruins as well as the cemetery with its mounds made of blocks of ironstone were visible again [55].

Furthermore, we recorded 14 locally used BaKongo proverbs that deal with the subject of death (S3 Table) but of which none refers to plants.

## Discussion

### Empty cemeteries?

Although more than 24% of the visited cemeteries lacked any planting such graveyards do not consist of bare soil only but personal belongings. This practice was already documented by Bastian (1874) [32], Pechuël-Loesche (1907) [35], or Laman (1957) [31]. All articles put on the graves are thought to have been used by the deceased (Figs 2B, 2E and 3A) [31]. This has been described for several Bantu tribes. The gifts are considered as a medium to communicate with the ancestors [56].

Furthermore, symbols made of wood or concrete are used to mark the site. These crosses, at first glance, seem to be Christian symbols. At a second glace, however, one may discover relations to the *dikenga dia kongo*. The cross in Fig 2F displays two characteristic arrangements of nails. However, the Greek cross (+) may serve as symbol for oath taking as well. The horizontal line represents the boundary; the vertical one ambivalently represents both the path leading across the boundary, and the vertical path of power linking "the above" with "the below". In this way, the person taking the oath stands upon the cross, situating himself between life and death [57]. The crossed lines therefore demarcate spiritual spaces [38] or places of judgment [35].

### Symbolism of the used plants

Traditionally, plants are not part of the BaKongo burial ceremonies. Nevertheless, our recent study showed that a variety of species, with a high percentage of alien species, are used. Especially in the urban municipality Uíge and the adjacent municipality Bungo that is crossed by the main road to the DRC the relative diversity of plant species occurring in the cemeteries is remarkable. This is most likely because Uíge city as the capital of the province was also the heart of the Portuguese administration as well as the religious center of the Catholic Church and the influence of European burial culture therefore was strong.

Considering plant species and their characteristics, north Angolan cemeteries do not show similarities with European ones (except in Uíge central cemetery) but some components exhibit an essential and historically founded relation to other areas, either in Africa or in other tropical areas of the world. For Euphorbiaceae earlier literature suggests the accentuated use for rituals [58], while for Asparagaceae no research addressing their ritual functions is available. Taking into account that the Asparagaceae species belonged to Agavaceae earlier on, the two families Agavaceae and Euphorbiaceae together account for 70% of the plants found on cemeteries. In the following, we will look at the 10 most frequently recorded species individually to highlight their relation to other cultures.

#### Euphorbiaceae

Euphorbiaceae normally contain toxic substances that protect the plant against herbivorous animals [59]. Therefore, they are well suited as living fences or objects on graveyards, which will not be eaten.

Euphorbia tirucalli *L*. According to our results, *Euphorbia tirucalli* (syn. *E*. *rhipsalioides*) is the most abundantly seen species. Ficalho (1947) already described its use for fencing by local people in Angola [60]. A first record of its ritual use is given by Gossweiler (1950) [54] who already described its occurrence in the interior of “the colony” [Angola]. He wrote that very well-developed individuals are especially found on locations abandoned by its former inhabitants because *Euphorbia tirucalli* was planted on graves. Latham et al. (2014) [43] confirmed its use in the adjacent Bas-Congo region in the DRC for grave decoration but also for hedges. In southern African countries, it is often used as fencing plant (Bandeira et al. 2006, Palgrave 1990) probably also to deter marauders [61,62].

Euphorbia *cf*. ingens *E*.*Mey*. *ex Boiss*. This species is the third most common plant. Interviewees attributed its importance to its capability to serve as a grave marker (Fig 3G). Cuttings root and establish quickly and, in addition, the name of the deceased can be engraved in the long lasting epidermal and sub-epidermal tissues. Gossweiler (1950) [54] did not report this species in Angola, which indicates that planting *E*. cf. *ingens* could be a rather modern decorative plant for cemeteries. On the other hand, it is native to southern and eastern African countries and therefore could have been introduced easily before the arrival of the Portuguese.

To confirm the identification definitely, further studies should be undertaken.

Euphorbia pulcherrima *Willd*. *ex Klotzsch*. The poinsettias, used because of their flaming red bracts, are less used for cemetery decoration than *E*. *tirucalli* and *E*. cf. *ingens*. In Western countries the plant is part of Christian symbolism and known as Easter flower or Santa Claus flower [63]. In the poem “Christmas Eve at Rousemound Cemetery” Susan Wood mentions the poinsettias on graves in the United States. It is mentioned for Angola by Gossweiler (1950) [54] as a decorative but only rarely found plant in the gardens of the Portuguese settlers.

Jatropha curcas *L*. The use of *Jatropha curcas* originating from the Americas for the planting of hedgerows in Angola was again documented by Ficalho (1947) [60] and Gossweiler (1950) [54]. It is also confirmed by Latham et al. (2014) [43] for the Bas-Congo. In the province Uíge it is often planted (Fig 2F).

#### Asparagaceae

Dracaena fragrans *(L*.*) Ker Gawl*. *Dracaena fragrans* is native to Angola´s north and, in the context of graveyards, represents the most important species of Asparagaceae in cemeteries. Sheridan (2008) [64] describes the ritual use of Dracaena in East Africa. Besides its use as living hedges, Dracaena is also used to mediate and resolve social conflicts. A sprig of *Dracaena* protects persons against attacks even in most severe conflicts. Sending a leaf to one's rival “requires them to listen to one's argument without anger or resistance” [65]. In the same study, he also pointed out the planting of *Dracaena* on gravesites in Tanzania, later turning them into small sacred groves, so called *mpungi*. Especially the rectangular Muslim graves are marked with *Dracaena* at each of its corners. In total, he compiled 38 articles of the use of *Dracaena* on graves and shrines in 14 African countries, from East to West Africa.

Cordyline fruticosa *(L*.*) A*.*Chev*. For America, Sheridan (2011) [65] found that *Cordyline fruticosa* takes the place of Dracaena for this purpose. Originating from Asia it has been broadly utilized as a ritual and sacred plant [66,67]. According to Sheridan (2011) [65] *Cordyline* was part of a “floating forest” by which plants from Tahiti were brought to the newly established botanical gardens in the Caribbean in the year 1791. Now it serves as “half boundary marker, half religion”. By contrast, van Andel et al. (2013) [3] stated that Javanese and East Indian migrants that came to Suriname to work on the plantations introduced their magical plants such as *Cordyline fruticosa*, which was planted on Javanese cemeteries and later became integral part in Afro-Surinamese religious ceremonies. A priest of a Maroon village in Surinam explained its meaning: “the shrub’s slender stem allows the dead person’s spirit to ascend easily into the sky, preventing it from staying on earth and troubling its family members” [3]. Neither Gossweiler (1950) [54] nor Latham et al. (2014) [43] mentioned this species for the area of the former Kongo Kingdom. Only Pauwels (1993) [44] described it as a decorative plant in gardens of the region Kinshasa-Brazzaville (DRC) due to its variegated leaves indicating a rather recent introduction to Uíge Province.

#### Other plant species

Agave sisalana *Perrine*. The production of sisal of about 60,000 tons per year was an important economic factor for the Portuguese until Angolan independence [54,68]. Although a symbol for slave labor, it is frequently found on cemeteries but as marker for limitations, too. Its characteristic inflorescence marks graveyards from larger distances. Furthermore, the spines at the leaf tips deter animals from eating them.

Elaeis guineensis *Jacq*. Palm leaves are deeply rooted in Christian culture and symbolize the victory of the faithful [69]. But even before that, palms were a symbol for victory, peace and eternal life in other cultures. In Uíge´s burial culture, *Elaeis guineensis* plays a major role (beside its even more important role as a food). The long leaves decorate funeral corteges, they are woven to garlands to put on the graves (Fig 2D) but primarily to braid beautiful entrances to the cemeteries in case of a funeral, perhaps reminders of the Christian tradition of waving palm branches at the triumphal entry of Jesus into Jerusalem. Muluwa et al. (2010) [39] mentioned the palm wine made of this species for the use in several ceremonies of the Mbuun, the Mpiin and the Nsong in the Bandundu Province (DRC) due to its high symbolic and ritual importance. To establish contact to the ancestors a certain quantity must be spread across the soil. This ritual is similar to those in Uíge Province where the palm wine of either *Elaeis guineensis* or *Raphia matombe* is used [39,70].

Catharanthus roseus *(L*.*) G*.*Don*. Foxcroft et al. (2008) [71] mentioned *Catharanthus roseus* as an invasive plant in the Kruger National Park, South Africa. Due to their investigations, it also bears the name “graveyard flower”.

#### Can a plant be considered as a Kongo Cosmogram?–symbolism of color and form

Völger (1990) [53] interpreted the significance of colors, symbols and gestures of the Niombo objects of the Babwende tribe but also of the Muzidi of the Babembe tribe, which were mainly collected by Laman [31,72–74]. Her interpretations were focused on the studies of the Zairean cultural scientist Fu-Kiau kia Bunseki and studies on colors of the Swedish ethnologist Jacobson-Widding. The main colors used for the Niombo figures are red, black and white. Black as symbol for the evil and white as symbol for the good are both used because death can be double-faced as well. In contrast, red is the color of the magic power and can definitely be seen as representing the characteristic dichotomy of the deceased. Beside the typical arm position, ornamental signs on the figures´ bodies are explained. Different lines run through the faces and abdomens, for instance vertical strokes from the eyes, symbolizing tears (mansanga), or a vertical line above the nose, symbolizing bravery when enduring pain (nkasunga). The most important sign is called “four cardinal points” and appears in various variations, even as a cross, which from a European or Christian perspective led to speculations on its origin. The spiral signifies the ongoing course of life and the transition between this and the next world.

Colors often have a symbolic representation. Hutchings (2004) [4], however, points to the economic driving force behind color selections such as the preference for black and white in mourning procedures in Europe and Asia as their colors are cheap and easy to clean. In other parts of the world (Mexico, Bali) bright colors are worn during funeral ceremonies. For West Africa red is associated with the protection from evil spirits and therefore of importance in funerals (Hutchings 2004) [4].

Taking the BaKongo cosmogram into account another dimension of color symbolism becomes apparent. The four daily positions of the sun symbolize the four life stages and are connected to each other by a circle. Kala, the childhood stage, symbolized by the sunrise, reflects the beginning of life on earth as well as joy and hope (symbol color black) followed by tukula, the adult stage, or noon. It represents the first animals on earth and the physical power of men (symbol color red). The next stage, luvemba, means old age and death, sunset and the first humans on earth (symbol color white). While the last stage is called musoni, reflecting midnight, spiritual power and the beginning of the earth (symbol color yellow) [75,76]. The land of the dead, in KiKongo called *nsi a bafwa* or *mpemba*, is represented by the color white as a symbol for purity, innocence and dead. In contrast, red and yellow symbolize the transformation and movement between the worlds [38,75]. Thus, corpses were often adorned in red. Looking at the colors represented on cemeteries in Uíge, red is the color used most, followed by yellow and white. The color blue was found just within one species. The dominance of red, yellow and white might be a hidden indicator for the cosmogram–nevertheless, the interpretation of the use of colors remains only speculation.

Colors are mainly features of flowers, less frequently of leaves and stems. Vickery (1984) [77] postulated that “flowers symbolize human mortality, and are equally symbolic of resurrection and rebirth, springtime and autumn, renewal and decay, and have long continued to provide consolation and hope at critical times in man's life.” People interviewed in this study put it this way “they are planted due to their beauty and therefore spring from heart”.

However, the most frequently found plants are just green though they showed another specific feature: either an iterative growth evoking the concept of self-similarity or a spiral growth of leaves along the shoot.

The spiral as a motif, often symbolized by a snail´s shell, is very complex. Eliade (1961) [78] establishes a connection to moon, lightning, water, fecundity, birth and life beyond the grave. Even in prehistoric cultures such as the Celts, spirals are found, emphasizing movement [79]. Martínez-Ruíz (2013) [25] describes the symbol with the attribute of movement. In our study, the spiral arrangement of leaves was found mainly in the mentioned monocotyledons (Liliopsida), especially in *Dracaena*, *Cordyline*, *Elaeis* and *Agave*. Here the plant´s growth pattern may augment the symbolic nature of a graveyard.

The “iterative forked branching”, reminding of fractal structures, can be seen as a simplified cross. For the BaKongo, crossroads and even a simple forked branch may represent the sacred [35]. We found this bifurcated branching in *Dracaena fragrans*, *Euphorbia tirucalli*, *E*. cf. *ingens* and *Jatropha curcas*, as well as in *Opuntia ficus-indica* L. Mill. On the other hand, self-similar patterns, as e.g. iterating branching, can be seen as symbols for eternity.

According to Laman (1957) [31] road forkings or junctions played an important role during burial ceremony of the BaKongo. The widow or widower did not escort the funeral procession to the graveyard but bade farewell to the deceased at the road forking and went back home. Later he/she had to sing at all road forkings that the corpse had been taken along and at the end of the song smashed a water-calabash [31].

### Alien plants

The high percentage of alien plant species among the plants used in graveyards was surprising but literature analyzed for comparison revealed similar results. Ajewole et al. (2015) [80] investigated eight different private and governmental cemeteries in Nigeria and found a variety of exotic plants as well, especially on private and religiously oriented cemeteries. The study of Muslim graveyards in Israel showed even more plant species from other regions [13]. Since exotic plants differ from the natural vegetation, especially if they are large and visually contrasting, they are well suited as markers, which may explain their frequent use on graveyards.

## Conclusion

BaKongo cemeteries traditionally do not exhibit plants for decoration purposes. Still our study conducted in northern Angola documented for the first time a large proportion of graves decorated with plants or plant parts, with a high number of alien species mainly from the Americas, which could be an indication of outside influence. Euphorbiaceae and Asparagaceae are the most important plant families, amounting to 70% of the species found.

Fennell (2007) [38] warns of interpreting symbols without care because African cultures can share the use of colors and lines. Moreover, in northern Angola a strong influence of Portuguese or rather Catholic authority can be observed. Nevertheless, our results showed a variety of different patterns in north Angolan cemeteries, which might be connected to the BaKongo symbolism. Symbols were not just found in components like wooden crosses but also in plants´ form and colors. A certain percentage of cemeteries though still remains empty or decorated with “traditional objects”.

To support the listed data we suggest collecting data via the accompanying of today´s burial ceremonies. These data may provide valuable insights into the influence of nature on rituals such as planting.

## Supporting information

S1 Questionary (DOCX)

S1 Table Species found on cemeteries in Uíge province. Species listed according to their frequency, starting with the most abundant. We added the following information: vernacular names and its translations, plant family, the origin of the species, the color of characteristic feature or a growth pattern, medicinal use [MU], number of cotyledons [CO], growth form [GF], the voucher number and the percentage of cemeteries the species was found. Origin: Af = Africa, Am = America, AS = Asia, Mad = Madagascar, * marks neophytes; Pattern: it = iterative growth, sp = spiral growth; Medicinal use (MU): 0 = no use, 1 = medicinal use according to Lautenschläger et al. (2018);Number of cotyledons (CO): 1 = Monocotyledons, 2 = Dicotyledons; growth form (GF): P = perennial, S = shrub, T = tree; Voucher: F = foto voucher, HD = Herbarium Dresdense.
(DOCX)

S2 Table Species listed according to the municipalities were they were found. KA = Kangola, AM = Ambuila, BE = Bembe, BU = Bungo, DA = Damba, KI = Kimbele, MA = Maquela do Zombo, MI = Milunga, MU = Mucaba, NE = Negage, PU = Puri, SO = Songo, UI = Uíge.
(DOCX)

S3 Table List of 14 Kikongo proverbs. Proverbs recorded during the field studies in Uíge Province, and its translations into Portuguese and English.
(DOCX)
