# Supplementary material for: Stories told by plants on graveyards in Northern Angola
Source: PLoS One. 2020 Aug 17;15(8):e0236941. doi: 10.1371/journal.pone.0236941 (PMC7430708; doi:10.1371/journal.pone.0236941)
Supplement: S1 Questionary — (DOCX) [file pone.0236941.s001.docx]

**Questionary**

Date:

Name of the village:

Coordinates:

Name of interviewee:

Age:

Gender:

1. What is the name of this plant in Kikongo? Does this name have a meaning? Can it be translated (into Portuguese?)
2. Does this plant have any other names?
3. In tradition, why did people plant it on the grave? For what reason?
4. In tradition, does this plant attract the spirits or repel the spirits?
5. Can this plant assist someone to speak with ancestors? Were offerings traditionally placed near this plant?
6. Have there been any recent changes in the plant that is used on graves?

**Supplementary Questions**

1. Is it planted during the burial ceremony or later on another special day (how many days later?)?
2. Is it planted at the head or foot of the grave (or just anywhere on the grave)?
3. Who is responsible to plant and care for the grave? (e.g. elders, relatives of the deceased, children, parents?)
4. Is there a special prayer, song or ceremony for the planting?
5. Is there a traditional story or song, which tells about this plant and its uses?
6. When people see this plant, does it always mean that there is a grave underneath?
7. In tradition, does this plant bring good luck?
8. Is it planted for protection (against enemies? thieves? evil influences? lightning?)
9. Do people avoid this plant when they see it near a pathway?
10. Is this plant used for fences of homesteads? Is this plant uses to mark the boundary of a farm?
11. Is this plant used to mark special places?
    e.g a shrine?
    e.g marketplace?
    e.g. crossroads?

e.g. palaver tree/ palaver place?

1. Are there any other uses for this plant e.g. medicinal?
2. Are there any other kinds of plants that are planted in your graveyard?
3. Is there a special plant chosen for the grave of a man? A woman? A child? A twin? A traditional priest (nganga)? A criminal? A suicide? A Person who died of a terrible disease? A witch/ sorcerer?
