# Supplementary material for: Stories told by plants on graveyards in Northern Angola
Source: PLoS One. 2020 Aug 17;15(8):e0236941. doi: 10.1371/journal.pone.0236941 (PMC7430708; doi:10.1371/journal.pone.0236941)
Supplement: S1 Table — Species listed according to their frequency, starting with the most abundant. We added the following information: vernacular names and its translations, plant family, the origin of the species, the color of characteristic feature or a growth pattern, medicinal use [MU], number of cotyledons [CO], growth form [GF], the voucher number and the percentage of cemeteries the species was found. Origin: Af = Africa, Am = America, AS = Asia, Mad = Madagascar, * marks neophytes; Pattern: it = iterative growth, sp = spiral growth; Medicinal use (MU): 0 = no use, 1 = medicinal use according to Lautenschläger et al. (2018);Number of cotyledons (CO): 1 = Monocotyledons, 2 = Dicotyledons; growth form (GF): P = perennial, S = shrub, T = tree; Voucher: F = foto voucher, HD = Herbarium Dresdense. (DOCX) [file pone.0236941.s002.docx]

**S1 Table.** **Species found on cemeteries in Uíge Province.**

| **species** | **vernacular name** | **plant family** | **origin** | **color/ pattern** | **MU** | **CO** | **GF** | **Voucher** | **ID** | **no. of cemeteries** |
| --- | --- | --- | --- | --- | --- | --- | --- | --- | --- | --- |
| *Euphorbia tirucalli* L. | Mbika, mbiku | Euphorbiaceae | Af | it | 1 | 2 | S | F_46 |  | 23 |
| *Agave sisalana* Perrine | Mbarambata | Asparagaceae | *Am | sp | 0 | 1 | P | F_81 |  | 22 |
| *Euphorbia* cf. *ingens* E.Mey. ex Boiss. | Kitondo, Dize, diza | Euphorbiaceae | Af | it | 0 | 2 | S | F_82, F_83 |  | 18 |
| *Dracaena fragrans* (L.) Ker Gawl. | Mundalandala | Asparagaceae | Af | sp, it | 0 | 1 | S | F_84 |  | 14 |
| *Euphorbia pulcherrima* Willd. ex Klotzsch |  | Euphorbiaceae | *Am | red | 0 | 2 | S | F_77 |  | 12 |
| *Elaeis guineensis* Jacq. | Ngazi, Nkula, Nsamba | Arecaceae | Af | sp | 1 | 1 | T | F_44 |  | 12 |
| *Jatropha curcas* L. | Mpuluka, Sinde dia nkaka | Euphorbiaceae | *Am | it | 1 | 2 | S | DR043845 | 1211879 | 11 |
| *Catharanthus roseus* (L.) G.Don |  | Apocynaceae | *Mad | pink white | 1 | 2 | P | DR043937 | 1208676 | 9 |
| *Caladium bicolor* (Aiton) Vent. | Malanga | Aracaeae | *Am | red white | 0 | 1 | P | F_79 |  | 7 |
| *Cordyline fruticosa* (L.) A.Chev. |  | Asparagaceae | *As | sp | 0 | 1 | S | F_86 |  | 6 |
| *Sansevieria* sp. | Lingua da sogra | Asparagaceae | Af |  | 0 | 1 | P | F_78 |  | 6 |
| *Stachytarpheta cayennensis* (Rich.) Vahl | Agua de joelho | Verbenaceae | Af | blue | 1 | 2 | P | DR042710 | 1262350 | 4 |
| *Amaryllis* sp. |  | Amaryllidaceae | Af | white | 0 | 1 | P |  |  | 3 |
| *Brugmansia aurea* Lagerh. |  | Solanaceae | *Am | yellow | 1 | 2 | S | F_87 |  | 2 |
| *Canna indica* L. | Chololo | Cannaceae | *Am | red | 1 | 1 | P | DR042701 | 1210707 | 2 |
| *Erythrophleum africanum* (Benth.) Harms | Ngungu | Fabaceae | Af |  | 1 | 2 | T | DR043233 | 1253603 | 2 |
| *Euphorbia cotinifolia* L. |  | Euphorbiaceae | *Am | red white | 0 | 2 | S | F_85 |  | 2 |
| *Opuntia ficus-indica* L. Mill. |  | Cactaceae | *Am | yellow | 0 | 2 | S | F_80 |  | 2 |
| *Senna occidentalis* (L.) Link | Manioka nioka, Mansambi nsambi nkau | Fabaceae | *Am | yellow | 1 | 2 | S | DR050121 | 1270722 | 2 |
| *Tecoma stans* Griseb. |  | Bignoniaceae | *Am | yellow | 0 | 2 | S |  |  | 2 |
| *Alchornea cordifolia* (Schumach. & Thonn.) Müll.Arg. | Mbunza | Euphorbiaceae | Af | red | 1 | 2 | T | DR042586 | 1211855 | 1 |
| *Cereus* sp. | Nsoma | Cactaceae | *Am | it | 0 | 2 | S | F_23 |  | 1 |
| *Lantana camara* L. |  | Verbenaceae | *Am | red yellow | 1 | 2 | P | DR043374 | 1262300 | 1 |
| *Newbouldia laevis* (P.Beauv.) Seem. ex Bureau | Kafuki, Kavuki, Kuvuiti | Bignoniaceae | Af |  | 1 | 2 | T | DR043913 | 1211985 | 1 |
| *Pachira glabra* Pasq. | Nguba | Malvaceae | *Am | white | 0 | 2 | T | DR056617 | 1471929 | 1 |
| *Tithonia diversifolia* (Hemsl.) A.Gray. | Malulua, Malulu | Asteraceae | *Am | yellow | 1 | 2 | P | DR044090 | 1210181 | 1 |
| *Vitex madiensis* Oliv. | Mafilu | Lamiaceae | Af |  | 1 | 2 | S | DR042773 | 1212133 | 1 |

Species listed according to their frequency, starting with the most abundant. We added the following information: vernacular names and its translations, plant family, the origin of the species, the color of characteristic feature or a growth pattern, medicinal use [MU], number of cotyledons [CO], growth form [GF], the voucher number and the percentage of cemeteries the species was found. Origin: Af = Africa, Am = America, AS = Asia, Mad = Madagascar, * marks neophytes; Pattern: it = iterative growth, sp = spiral growth; Medicinal use (MU): 0 = no use, 1 = medicinal use according to Lautenschläger et al. (2018);Number of cotyledons (CO): 1 = Monocotyledons, 2 = Dicotyledons; growth form (GF): P = perennial, S = shrub, T = tree; Voucher: F = foto voucher, DR = Number in Herbarium Dresdense, ID = specimen ID in Virtual Herbaria JACQ.
