# Supplementary material for: Stories told by plants on graveyards in Northern Angola
Source: PLoS One. 2020 Aug 17;15(8):e0236941. doi: 10.1371/journal.pone.0236941 (PMC7430708; doi:10.1371/journal.pone.0236941)
Supplement: S2 Table — KA = Kangola, AM = Ambuila, BE = Bembe, BU = Bungo, DA = Damba, KI = Kimbele, MA = Maquela do Zombo, MI = Milunga, MU = Mucaba, NE = Negage, PU = Puri, SO = Songo, UI = Uíge. (DOCX) [file pone.0236941.s003.docx]

**S2 Table.** **Species listed according to the municipalities were they were found.**

| **species** | **KA** | **AM** | **BE** | **BU** | **DA** | **KI** | **MA** | **MI** | **MU** | **NE** | **PU** | **SO** | **UI** | **TOTAL** |
| --- | --- | --- | --- | --- | --- | --- | --- | --- | --- | --- | --- | --- | --- | --- |
| *Euphorbia tirucalli* |  |  |  |  | 12 |  | 5 |  |  |  |  | 1 | 3 | 20 |
| *Agave sisalana* | 1 | 1 |  | 1 | 7 |  | 2 |  | 2 | 2 | 1 |  | 2 | 18 |
| *Euphorbia* cf. *ingens* |  |  | 2 | 1 | 4 |  |  |  | 1 | 2 |  | 2 | 3 | 14 |
| *Dracaena fragrans* |  |  |  | 2 |  |  | 1 | 1 | 1 | 3 |  | 1 | 4 | 12 |
| *Euphorbia pulcherrima* |  |  |  | 1 | 1 |  |  |  |  | 4 | 1 | 1 | 2 | 10 |
| *Elaeis guineensis* |  |  |  | 1 | 2 |  |  |  | 2 | 1 | 2 |  | 2 | 9 |
| *Jatropha curcas* |  | 2 | 1 |  |  |  |  |  | 1 | 2 |  | 2 | 1 | 9 |
| *Catharanthus roseus* |  | 1 | 2 |  |  |  | 1 |  |  |  |  | 2 | 1 | 7 |
| *Caladium bicolor* |  | 2 | 2 |  |  |  |  |  |  |  |  |  | 2 | 6 |
| *Cordyline fruticosa* |  |  |  |  |  |  |  |  |  | 2 |  | 1 | 2 | 5 |
| *Sansevieria* sp. |  |  | 2 |  |  |  |  |  |  |  |  | 1 | 2 | 5 |
| *Stachytarpheta cayennensis* |  |  |  |  | 3 |  | 1 |  |  |  |  |  |  | 4 |
| *Amaryllis* sp. |  |  |  |  |  |  |  |  |  | 1 |  |  | 2 | 2 |
| *Brugmansia aurea* |  |  |  |  |  |  |  |  |  | 1 |  |  | 1 | 2 |
| *Canna indica* |  |  |  |  |  |  |  |  |  | 1 |  |  | 1 | 2 |
| *Erythrophleum africanum* | 2 |  |  |  |  |  |  |  |  |  |  |  |  | 2 |
| *Euphorbia cotinifolia* |  |  |  |  |  |  |  |  |  |  |  | 1 | 1 | 2 |
| *Opuntia ficus-indica* | 1 |  |  |  |  |  |  |  |  |  |  | 1 |  | 2 |
| *Senna occidentalis* |  |  |  | 2 |  |  |  |  |  |  |  |  |  | 2 |
| *Tecoma stans* |  |  | 1 |  |  |  | 1 |  |  |  |  |  |  | 2 |
| *Alchornea cordifolia* |  |  |  |  |  |  |  |  |  |  |  |  | 1 | 1 |
| *Cereus* sp. |  |  |  |  |  |  |  | 1 |  |  |  |  |  | 1 |
| *Lantana camara* |  | 1 |  |  |  |  |  |  |  |  |  |  |  | 1 |
| *Newbouldia* *laevis* |  |  |  |  |  |  | 1 |  |  |  |  |  |  | 1 |
| *Pachira glabra* |  |  |  |  |  |  |  |  |  | 1 |  |  |  |  |
| *Tithonia diversifolia* |  |  |  | 1 |  |  |  |  |  |  |  |  |  | 1 |
| *Vitex madiensis* |  |  |  |  |  |  |  |  |  |  |  | 1 |  | 1 |
| No. of species | 3 | 5 | 6 | 7 | 6 | 0 | 7 | 2 | 5 | 11 | 3 | 11 | 16 | 79 |
| No. of visited cemeteries | 4 | 5 | 4 | 2 | 22 | 2 | 15 | 1 | 7 | 5 | 8 | 8 | 4 | 86 |
| No. species/no. cemeteries | 0.8 | 1 | 1.5 | 3.5 | 0.3 | 0 | 0.5 | 2 | 0.7 | 1.8 | 0.5 | 1.4 | 4 | 0.9 |

KA = Kangola, AM = Ambuila, BE = Bembe, BU = Bungo, DA =Damba, KI = Kimbele, MA = Maquela do Zombo, MI = Milunga, MU = Mucaba, NE = Negage, PU = Puri, SO = Songo, UI = Uíge.
