# Supplementary material for: Stories told by plants on graveyards in Northern Angola
Source: PLoS One. 2020 Aug 17;15(8):e0236941. doi: 10.1371/journal.pone.0236941 (PMC7430708; doi:10.1371/journal.pone.0236941)
Supplement: S3 Table — Proverbs recorded during the field studies in Uíge Province, and its translations into Portuguese and English. (DOCX) [file pone.0236941.s004.docx]

**S3 Table.** **List of 14 Kikongo proverbs**.

| **Kikongo proverb** | **Portuguese translation** | **English translation** |
| --- | --- | --- |
| Ya fwa nki nsisa? Yazinga nki dia? | Se eu morrer, o que deixarei? Se viver ainda, o que comerei? | If I die, what will I leave? If I still live, what will I eat? |
| Ekolo tat aka fwidiko, zaya ntalu a kwanga. | Enquato o pai ainda nao morreu, saiba (já) o preço de kwanga. | While ones’ parents are still alive, learn how to fight for your life. |
| Ngudi aku kani ka fweko, longoka kunga wa dilu. | Enquanto a tua mae ainda nao morreu, aprende o cantico de chorar. | As long as your mother is still alive you should learn the dirge. |
| Mvumbi wa fwa kia meso, kansi ka fwa kia matuko. | Os cadáveres perdem a visão,  nunca à audição. | The eyes of a corpse die, but not its ears. |
| Tufwanga mu soba. | Morrer não é o fim. | Dying is not the end. |
| Tata ka fwa ko. Fubwa kafubwa | O pai não morreu, mas foi queimado. | The father did not die but was burned. |
| Mpese fwidi mu mwamba, kiese kiandi kididi. | A barata que morreu no molho, foi vítima da sua alegria de comer. | The cockroach which dies in the sauce was victim of its joy to eat. |
| Mboma ka fwe, maki ka ma fwe | Que não morre a jiboa nem se estrague os ovos. | As long as the serpent does not die the eggs are not fouled. |
| Disu di fuidi fuku betela ye tulu | A vista que estragou de noite coincidiu com o sono. | The view that was lost at night coincided with sleep |
| O fwa yi tu fuidi yandi gwola yi tu tina? | Se já morremos será que vamos fugir de podrecer? | If we are dead is it possible to flee the decay? |
| Mfwilwa n´tete, ki fwilwa ngomba ko. | Qué me morra o cesto e nao o trabalhador criado. | The basket can break but not the one who made it. |
| Tata ka fwa ko, kansi mu nzo ka vila. Evia ko nkuankake. | O pai nao morreu, más foi queimado dentro da casa. Será que a morte difere-se de ser queimado. | The father did not die but was burned inside the house. Is it possible that death differs from being burned? |
| Ba fwa ba tondua | Os mortos sao sempre louvados | The dead are honoured forever. |
| Vuna fwa wazaya bakuzolanga | Finge-se de morrer para observar, conhecer ou saber os que te amam | Feign your death to find out who really loves you. |

Proverbs recorded during the field studies in Uíge Province, and its translations into Portuguese and English.
